# In Vitro Enzyme Inhibitory Properties, Secondary Metabolite Profiles and Multivariate Analysis of Five Seaweeds

**DOI:** 10.3390/md18040198

**Published:** 2020-04-08

**Authors:** Mohamad Fawzi Mahomoodally, Nabeelah Bibi Sadeer, Gokhan Zengin, Zoltán Cziáky, József Jekő, Alina Diuzheva, Kouadio Ibrahime Sinan, Kishneth Palaniveloo, Doo Hwan Kim, Kannan R. R. Rengasamy

**Affiliations:** 1 Institute of Research and Development, Duy Tan University, Da Nang 550000, Vietnam; mohamadfawzimahomoodally@duytan.edu.vn; 2 Department of Health Sciences; Faculty of Science, University of Mauritius, Réduit 80837, Mauritius; nabeelah.sadeer1@umail.uom.ac.mu; 3 Department of Biology, Science Faculty, Selcuk University, 42250 Konya, Turkey; gokhanzengin@selcuk.edu.tr (G.Z.); sinankouadio@gmail.com (K.I.S.); 4 Agricultural and Molecular Research and Service Institute, University of Nyíregyháza, 4400 Nyíregyháza, Hungary; cziaky.zoltan@nye.hu (Z.C.); jjozsi@gmail.com (J.J.); 5 Faculty of Forestry and Wood Sciences, Czech University of Life Sciences, 16500 Prague, Czech Republic; adyuzheva@gmail.com; 6 Institute of Ocean and Earth Sciences, Institute for Advanced Studies Building, University of Malaya, Wilayah Persekutuan Kuala Lumpur 50603, Malaysia; kishneth@um.edu.my; 7 Department of Bioresources and Food Science, Konkuk University, Seoul 05029, Korea; kimdh@konkuk.ac.kr

**Keywords:** seaweeds, antioxidants, tyrosinase, bioactive metabolites, biological activities

## Abstract

Seaweeds have been exploited as both food products and therapeutics to manage human ailments for centuries. This study investigated the metabolite profile of five seaweeds (*Halimeda* spp., *Spyridia hypnoides* (Bory de Saint-Vincent) Papenfuss, *Valoniopsis pachynema* (G. Martens) Børgesen, *Gracilaria fergusonii* J. Agardh and *Amphiroa anceps* (Lamarck) Decaisne using ultra-high-performance liquid chromatography coupled with electrospray ionization mass spectrometry (UHPLC-ESI-MS/MS). Furthermore, these seaweeds were assessed for antioxidant and inhibitory effects against α-amylase, α-glucosidase, acetyl-cholinesterase (AChE), butyryl-cholinesterase (BChE) and tyrosinase. *Valoniopsis pachynema* and *A. anceps* yielded the highest flavonoid (4.30 ± 0.29 mg RE/g) and phenolic content (7.83 ± 0.08 mg RE/g), respectively. Additionally, *A. anceps* exhibited significant antioxidant properties with all assays and significantly depressed BChE (IC_50_ = 6.68 ± 0.83 mg/mL) and α-amylase activities (IC_50_ = 5.34 ± 0.14 mg/mL). Interestingly, the five seaweeds revealed potent inhibitory effects against tyrosinase activity. In conclusion, *A. anceps* might be considered as a key source of phytoantioxidants and a potential candidate to develop nutritional supplements. Besides, the five tested seaweeds warrant further study and may be exploited as promising natural sources for managing hyperpigmentation.

## 1. Introduction

Seaweeds are the ‘lungs of the sea’ as well as a potential ‘wild pharmacy’. These plant like organisms produce 70–80% oxygen for the atmosphere and possess scads of metabolites with unique structures of medicinal values [1,2]. In addition to their ecological importance, seaweeds have been a source of food for humans since ancient times. Seaweeds, also referred as algae, are commonly consumed as either fresh or dried in Asian, African and European countries. The consumption of seaweeds as food products can be traced back to the fourth century in Japan. Seaweeds are rich in vitamins A, E, C, B_1_ and B_12_, carbohydrates and organic iodine. The annual human consumption of algae in a dried form is estimated to be 2,000,000 tons [3]. In addition to their nutritive value, seaweeds are known to possess a wide array of valuable pharmacological properties including use as antibiotics, anticoagulants, antiulcer, antioxidants, antimicrobials, and antifouling [4,5]. 

Nonetheless, there is still a dearth of scientific data on the pharmacological and chemical profiles of seaweeds. Therefore, the present study was designed to investigate the pharmacological and chemical profiles of five seaweeds originating from different families, namely *Halimeda* spp. (Family: Halimedaceae)*, Spyridia hypnoides* (Bory de Saint-Vincent) Papenfuss (Family: Spyridiaceae), *Valoniopsis pachynema* (G. Martens) Børgesen (Family: Valoniaceae), *Gracilaria fergusonii* J. Agardh (Family: Gracilariaceae) and *Amphiroa anceps* (Lamarck) Decaisne (Family: Lithophyllaceae) collected in Tamil Nadu, India. *Halimeda* is a well-known green algae made up of discs containing calcium carbonate [6]. Works of literature reported that *Halimeda* spp. has potential apoptosis, anti-inflammatory, antioxidant, neuroprotective and hepatoprotective properties [7]. *Spyridia hypnoides*, belonging to the family Spyridiaceae, is a 15 cm tall plant like organism with numerous branches at short intervals. No reproductive structures were observed in this seaweed [8]. Sudharsan et al. reported that the galactans isolated from *S. hypnoides* exhibited anticoagulant and antioxidant properties. *V. pachynema,* also known as AstroTurf algae, originates from the family Valoniaceae. It is a filamentous alga, spongy and tends to cover completely the surface (dead corals, rocks) on which it grows to form a ball-like appearance [9]. A study conducted by Kumar et al. reported high level of calcium (476.67 ± 6.2%) in this seaweed species [10]. *Gracilaria,* originating from Gracilariaceae family is often a source of food for many people in Malaysia [11]. The aqueous extract of *G. fergusonii* displayed anti-inflammatory activity at a dosage of 250 µg/ml with a percentage inhibition of 63.98% [12]. On the other hand, *A. anceps* originating from Lithophyllaceae family, is a red macroalga usually found in sea waters at temperatures of 5 to 15 °C. This seaweed was screened for its antagonistic activity. Data collected showed that the crude extract exhibited clear inhibition zones against several pathogens: *Yersinia* spp., *Streptococcus* spp. and *Vibrio* spp. [13]. However, since the existing literature is insufficient, fragmented and unsystematic, we embark on this present research to try to expand the currently limited literature.

A series of enzymes were chosen based on the current challenging diseases globally such as diabetes mellitus (DM) type II, Alzheimer’s disease and skin disorders. Enzymes play a considerable role in biological reactions, contributing a diversification platform to the pharmaceutical industry. There is a wide spectrum of applicability of enzymes in the pharmaceutical industry starting from nutraceuticals, enzyme therapy, disease diagnosis, to drug synthesis [14]. Herein, enzymatic inhibitions involving α-amylase and α-glucosidase were investigated for DM type II, acetyl- (AChE) and butyryl-cholinesterase (BChE) for Alzheimer’s disease, and tyrosinase for skin disorders. Furthermore, dysfunction of the antioxidant defensive system leads to the development of chronic health conditions related to degenerative pathologies such as cardiovascular diseases, cancer, and neurodegeneration disorders [15]. Thus, to prevent health complications, the body must rely on exogenous antioxidants to effectively suppress reactive oxygen species (ROS). Since seaweeds are widely consumed, it was indeed a matter of great interest for us to investigate their antioxidant properties as well.

This work was undertaken to encompass the following objectives—(1) conduct a quantitative estimation of phytochemicals using in vitro standard chemical assays and identify the compounds using ultra-high-performance liquid chromatography coupled with an electrospray ionization mass spectrometry (UHPLC-ESI-MS/MS) technique, (2) report the antioxidant capacities in terms of radical scavenging, reducing potential, metal chelating and determine the total antioxidant capacity, (3) evaluate the enzymatic inhibitory effects against clinical enzymes associated with chronic diseases, namely diabetes mellitus (α-amylase and α-glucosidase), Alzheimer’s disease (AChE and BChE) and skin hyperpigmentation (tyrosinase) and (4) analyse the collected scientific data using multivariate analysis.

## 2. Results and Discussion

### 2.1. Antioxidant Assays

As a normal protective mechanism, the human body naturally responds to oxidative stress (caused by reactive oxygen species (ROS)) using its antioxidant defence. Nevertheless, in some cases, the enzymatic systems fail to resist to ROS, and the level of antioxidants present is insufficient to successfully ascertain healthy cellular homeostasis [16,17]. The antioxidant properties of phytochemicals are hidden behind their ability to donate electrons and/or chelate metals without them being transformed into harmful radicals [18,19]. The search for potential antioxidant activities from marine sources is still not widespread [20]. Thus, to try to fill this niche, we screened the different extracts of each seaweed for their antioxidative properties. 

Considering the complexity of phytochemicals, multiple assays targeting different mechanisms of action were selected to assess antioxidant properties. For instance, radical scavenging was assessed by 2,2-diphenyl-1-picrylhydrazyl (DPPH) and 2,2′-azinobis-(3-ethylbenzothiazoline-6-sulfonic acid (ABTS), reducing power by ferric reducing power antioxidant (FRAP) and cupric reducing antioxidant capacity (CUPRAC), total antioxidant capacity by phosphomolybdenum (PHPD) and metal chelating by ferrous ion chelating assay. Results are summarized in Table 1. Overall, the methanolic extract of *A. anceps* displayed the strongest antioxidant properties with all assays and the least potent was revealed to be methanolic extract of *Halimeda* spp. Existing work in literature reported that phenolic compounds are one of the most effective antioxidants [21]. Indeed, this fact was consistent with our study since *A. anceps* possessed the highest amount of phenolic content explaining its high antioxidant capacities. Likewise, a study suggested that *A. anceps* could be an effective source of antioxidants considering its high level of algal phenolic compounds [22]. 

Metals are entwined in our body in a very complex way. They help in the normal functioning of the body; however, an imbalance in the level of metals can lead to health complications. For instance, an excess of iron reduces hepatic extraction and metabolism of insulin leads to peripheral hyperinsulinemia causing an increase in oxidative stress which affects the normal function of insulin, hence resulting in an increase in blood glucose level [23]. Thus, iron chelation could be an effective therapeutic approach. As shown in Table 1, the ferrous ion chelating ability decreased in the following order: *A. anceps* > *V. pachynema > S. hypnoides > Halimeda* spp. > *G. fergusonii*, with the latter showing no activity. Radical scavenging activity estimations of the five seaweeds were evaluated using DPPH and ABTS radicals due to their simplicity, sensitivity, speed, stability of the radicals and cheap instrumentation [24]. Results from the assays showed that the methanolic extract of *A. anceps* was the best DPPH and ABTS scavenger (4.43 ± 0.07 and 18.56 ± 0.10 mg TE/g, respectively) while the extract of *Halimeda* spp. displayed the least scavenging effect with both DPPH and ABTS radicals (2.33 ± 0.04 and 5.36 ± 0.47 mg TE/g, respectively). 

Furthermore, data gathered in this study showed that the crude methanolic extract of *A. anceps* possessed the most potent reducing power towards both Fe (III) and Cu (II) with Trolox equivalent values of 13.99 ± 0.30 and 46.47 ± 1.60 mg TE/g, respectively. However, *G. fergusonii* extract displayed weaker reducing potential against Cu (II) with 7.31 ± 0.07 mg TE/g, and *Halimeda* spp. extract was least potent on the reduction of Fe (III) with 4.91 ± 0.05 mg TE/g. The quantitative determination of total antioxidant capacity is based on the reduction of Mo (VI) to Mo (V) by the extract to form a green phosphate/Mo (V) complex under acidic condition [25]. Again, *A. anceps* extract with values of 0.73 ± 0.06 mmol TE/g demonstrated the optimal antioxidant capacity succeeded by *S. hypnoides* extract (0.37 ± 0.02 mmol TE/g), *V. pachynema* extract (0.30 ± 0.04 mmol TE/g), *G. fergusonii* extract (0.23 ± 0.01 mmol TE/g) and *Halimeda* spp. extract (0.21 ± 0.01 mmol TE/g) (Table 1). 

### 2.2. Enzymatic Inhibitory Properties

The World Health Statistics 2019, an annual compilation of the World Health Organization (WHO), stated that the world is constantly under health challenges with 40 leading causes of death including ischemic heart disease, Alzheimer’s disease, lung, liver, stomach, oesophagus and prostate cancer, chronic obstructive pulmonary disease, and stroke, among others [26]. The world is unhealthy, and our existing medications are either insufficient or ineffective. Thus, our fight against these chronic pathologies should be ongoing with new strategies on how to manage these diseases. One of the strategies currently considered by many researchers is to search for potent and more effective medications from plants. Currently, the marine ecosystem is a crucial source of medicinally important metabolites with pharmaceutical importance [27]. This present work is considered as second-to-none since we have screened the methanolic crude extracts of five different seaweeds, namely *Halimeda* spp., *S. hypnoides, V. pachynema, G. fergusonii* and *A. anceps* with regard to five key enzymes involved in chronic health complications. Inhibition of enzymes may be considered as a therapeutic approach; for instance, acetyl- (AChE) and butyryl-cholinesterase (BChE) inhibition: Alzheimer’s disease, α-amylase and α-glucosidase inhibition: diabetes mellitus, and tyrosinase inhibition: skin disorders. 

Results are summarized in Table 2. In terms of enzymatic properties, *A. anceps* extract significantly depressed BChE activity (IC_50_ = 6.68 ± 0.83 mg/mL) but weakly inhibited AChE activity (IC_50_ = 3.90 ± 0.83 mg/mL). Instead, the seaweed *Halimeda spp.* crude extract exhibited higher BChE inhibitory effect with IC_50_ value of 3.07 ± 0.10 mg/mL. AChE catalyses the hydrolysis of acetylcholine (ACh) into acetic acid and choline while BChE is responsible for ACh homeostasis [28]. However, an accumulation of ACh in synapses may result in muscarinic and nicotinic toxicity which subsequently cause muscle cramps, blurry vision, lacrimation, muscular weakness, and paralysis [29]. From this perspective, it is important to maintain a healthy ACh homeostasis in the body. Interestingly, it is noteworthy to highlight that BChE activity increases with the severity of dementia, thus from this background information it can be stated that searching for potent BChE inhibitors is of utmost importance, as demonstrated by the extracts of *Halimeda spp.* and *A. anceps* [30]. 

Diabetes is on the rise; since 1980, the number of patients with diabetes has increased four-fold to 422 million people irrespective of their age and gender [31]. Thus, there is still an urgent need to develop more efficient medications with lesser side effects. Results collected herein reported that the crude extract of *A. anceps* showed similar inhibitory effects against α-amylase (IC_50_ = 5.34 ± 0.14 mg/mL) and α-glucosidase (IC_50_ = 5.64 ± 1.19 mg/mL). On the other hand, *V. pachynema* extract demonstrated a higher inhibition against α-glucosidase (IC_50_ = 2.57 ± 0.02 mg/mL) in contrast to α-amylase (IC_50_ = 7.02 ± 0.28 mg/mL). Interestingly, pronounced inhibition of α-glucosidase is usually preferred over moderate α-amylase inhibition to develop antihyperglycemic agents with lesser gastrointestinal discomfort due to undigested carbohydrates [32]. 

The inhibitory effects of *Halimeda spp.*, *S. hypnoides, V. pachynema, G. fergusonii* and *A. anceps* extracts on tyrosinase enzyme were also evaluated. Tyrosinase is a copper-containing enzyme responsible for the process of converting l-tyrosine and 3,4-dihydroxyphenylalanine (l-DOPA) to melanin through the formation of dopaquinone. It is reported that excessive production of dopaquinone in the brain causes neurodegeneration and cell mortality. These disorders are the primary cause and the hallmark of Parkinson’s and Huntington’s disease [33]. Inhibition of tyrosinase may be considered as a therapeutic approach for managing skin disorders and more complicated diseases. Our present study showed that the crude methanolic extracts of the five seaweeds displayed relatively the same tyrosinase activity. For instance, the extract of *V. pachynema* exhibited IC_50_ value of 3.68 ± 0.03 mg/mL, *Halimeda spp.*: 3.70 ± 0.06 mg/mL, *S. hypnoides*: 3.73 ± 0.04 mg/mL, *G. fergusonii* 3.7. ± 0.05 mg/mL and *A. anceps:* 4.49 ± 0.15 mg/mL.

### 2.3. Bioactive Composition

The ongoing quest to discover novel natural bioactive compounds remains of utmost importance for a world which is currently facing multiple health challenges. It is highly acknowledged that plants, culinary herbs or even spices are incorporated with a myriad of phytochemicals possessing medicinal values. Phytoconstituents are known to minimize the risk of chronic and inflammatory conditions [34]. Interestingly, a recent review compiled by Gnanavel et al. reported that more than 15,000 bioactive metabolites have been identified from marine sources and seaweeds are among them [35]. Among the different classes of phytochemicals, polyphenols or phenolic compounds and flavonoids are the two most widely studied classes of bioactive compounds [36]. Thus, in our present study, we screened the investigated seaweed extracts *Halimeda* spp., *S. hypnoides*, *V. pachynema*, *G. fergusonii* and *A. anceps* for phenolic and flavonoid contents. 

Among the tested seaweeds, *A. anceps* possessed the highest phenolic content (7.83 ± 0.08 mg GAE/g) while *Halimeda* spp. yielded the lowest amount (2.24 ± 0.06 mg GAE/g). In terms of flavonoid content, *V. pachynema* yielded the highest amount, succeeded by *A. anceps*, *S. hypnoides*, *Halimeda* spp. and *G. fergusonii* (4.30 ± 0.29, 2.47 ± 0.22, 2.45 ± 0.16, 1.74 ± 0.08 and 0.42 ± 0.04 mg RE/g, in order of magnitude). 

To have an overview of the chemical diversity possessed by each seaweed, an extensive profiling technique, ultra-high-performance liquid chromatography coupled with electrospray ionization mass spectrometry (UHPLC-ESI-MS/MS), was used. The results are summarized in Table 3. Results showed that *A. anceps* possessed a greater diversity of compounds (23) in contrast to *V. pachynema* (19), *G. fergusonii* (11), *S. hypnoides* (10) and *Halimeda* spp. (9). However, three compounds remain unidentified in *A. anceps* and two in *Halimeda* spp. 

In the nontargeted analysis, we tried to identify all components present in the samples using UHPLC-ESI-MS/MS. Using HPLC-single stage Orbitrap MS, a broad spectrum of chemical classes could be separated and detected within 70 minutes. High resolution (35000) and high mass accuracy (<5 ppm) enabled identification of most compounds. Structural identification and characterization were carried out on the comparisons of their chromatographic and ESI-MS/MS data (retention time, exact mass and fragmentation pathway) with the corresponding standards and data reported in the previous literature. Loliolide, phenolic acids, C9-C20 carboxylic acids, amino acids and their derivatives, terpenes were identified in the extracts, but their number was quite different.

#### 2.3.1. *Halimeda* spp.

Three carboxylic acids were detected in negative ion mode. Compounds with a retention time of 13.50 had [M − H]^−^ ion at *m/z* 201.11268 (C_10_H_18_O_4_) suggesting one unsaturated bond in the aliphatic chain and they yielded a characteristic fragment ion at *m*/*z* 183.1017 corresponding to neutral loss of one water molecule (Table 3). 3-Methyladipic acid with a retention time of 17.32 is a well-known and characterized compound. Carboxylic acid with a retention time of 19.73 exhibited a molecular ion [M − H]^−^ at *m*/*z* 214.93438 (Figure S1 in the Supplementary Information). The isotopic pattern indicated the presence of one bromo atom in the compound and a fragment ion [M – H − 44]^−^ at *m*/*z* 170.9440 corresponding to loss of CO_2_. Two monoterpenoid lactones: loliolide and isololiolide were detected in positive ion mode [M + H]^+^ at *m/z* 197.11777. Their structures were identified by comparison of their mass spectra with previously reported values. Ions at *m/z* 179.1069 and *m/z* 111.92 confirmed the loss of H_2_O [M + H − H_2_O]^+^, and also the loss of the ring adjacent to the lactone [M + H − H_2_O − C_5_H_8_]^+^. In addition to caulerpin, we have detected another compound at *m/z* 399.13449, but the fragmentation of this unidentified alkaloid was different from caulerpin.

#### 2.3.2. *Spyridia hypnoides*

In this species, we have identified several well-known and characterized compounds, for example, pantothenic acid, 3-phenyllactic acid, riboflavin or lumichrome, loliolide and isololiolide were also present in this species (Table 3).

#### 2.3.3. *Valoniopsis pachynema*

We also detected loliolide and isololiolide in this species. In addition to some known compounds, we detected two carboxylic acids with retention times 35.38 and 35.87 (Table 3). These compounds had the same [M − H]^−^ molecular ions at *m/z* 201.14907 (C_11_H_22_O_3_) (Figures S2 and S3 in the Supplementary Information). In the MS2 spectrums we did not find water losses suggesting the presence of omega hydroxyl group or ether bond. We tentatively identified these two compounds as ω-hydroxyundecanoic acid isomers. In the case of compound 19 in Table 3 the situation was the same. We did not find water loss and the main fragment was *m/z* 59.0123 (CH_3_-COO^−^), so we tentatively identified this carboxylic acid as a ω-hydroxydodecanoic acid isomer.

#### 2.3.4. *Gracilaria fergusonii*

In this species, we have identified several compounds that we have already found in the previous algae. Besides these compounds, we have identified a terpene with molecular ion at *m/z* 199.13342 in the positive ionisation mode (Table 3). The fragmentation of this molecule was very similar to loliolide and isololiolide, but the compound and most of its fragments had two hydrogen atoms more related to loliolide. Based on the exact molecular mass and the similarity of the fragmentation to loliolide, we tentatively identified this compound as dihydrololiolide or dihydroisololiolide.

#### 2.3.5. *Amphiroa anceps*

In addition to some known and characterized compounds, we have detected three terpenes with molecular ions *m/z* 181.12285 (C_11_H_16_O_2_), 199.13340 (C_11_H_18_O_3_) and 335.22223 (C_20_H_30_O_4_), respectively, in the positive ionisation mode (Figure S4 in the Supplementary Information). Compound 18 yielded characteristic fragment ions at *m/z* 163.1119 and 145.1014 corresponding to neutral loss of two water molecules. Compounds 19 and 20 yielded characteristic fragment ions at *m/z* 181.1224, 163.1117, 145.1013 and 317.2114, 299.2008, 281.1904, respectively, matching a neutral loss of three water molecules (Table 3).

### 2.4. Multivariate Analysis

**Figure 1:**
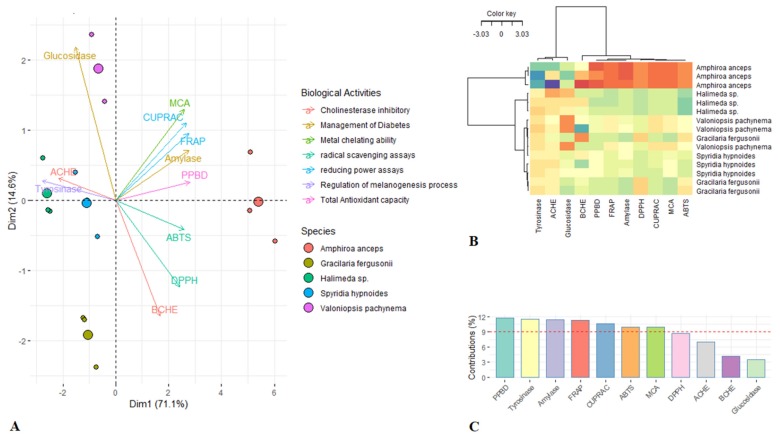
Multivariate analysis outcomes. (**A**) Score plot of multilevel the principle component analysis (PCA) model on the first two principal components. (**B**) Clustered image map based on the use of “Euclidean” distance and “Ward” linkage method. (**C**) Biological activities discriminating the species as gained by the evaluation of the relation between the 11 studied biological activities and the first component of the PCA.

The unsupervised multivariate analysis, principal component analysis (PCA) and Hierarchical cluster analysis (HCA) were used to comprehensively screen trends or resemblance between samples, i.e., whether they clustered according to the evaluated biological activities and to identify the key biological activities that contribute to the explanation of most the variance in the dataset. PCA aims to reduce the dimensionality of the data through summarizing as much information as possible. As shown in the PCA score plot, *A. anceps* was effectively discriminated from the other species (*V. pachynema*, *G. fergusonii*, *Halimena* spp., and *S. hypnoides*) (Figure 1A). In more detail, this segregation was done along the first component that was defined as the linear combination of seven biological activities (PPBD, tyrosinase, ABTS, CUPRAC, FRAP, metal chelating ability (MCA), α-amylase) (Figure 1C). Alongside this, the clustered image map (CIM) displayed a good classification of the samples into two distinct groups (Figure 1B). CIM was based on the hierarchical clustering simultaneously operating on the use of “Euclidean” distance and “Ward” linkage method. The HCA result was consistent with the principal component analysis, indicating the seven biological activities mentioned above effectively characterize the differences between *A. anceps* and the other species. Additionally, biological activities recorded for *A. anceps* were most potent among all the studied seaweeds, suggesting *A. anceps* as the most bioactive species. 

## 3. Materials and Methods 

### 3.1. Materials and Extraction

The five seaweeds, namely *Halimeda* spp., *Spyridia hypnoides* (Bory de Saint-Vincent) Papenfuss, *Valoniopsis pachynema* (G. Martens) Børgesen, *Gracilaria fergusonii* J. Agardh and *Amphiroa anceps* (Lamarck) Decaisne were collected from Mandapam coast, Gulf of Mannar, Tamil Nadu, India during March 2018. The collected seaweeds were identified by Dr. R. Arumugam, Department of Botany, A. V. C. College, Mannampanthal, Tamil Nadu, India. The collected sample was brought to the laboratory and washed thoroughly with tap water to remove all the extraneous materials and shade dried. The extracts were prepared as described in a previous study [37]. 

### 3.2. Determination of Antioxidant and Enzyme Inhibitory Effects

The radical scavenging (1,1-diphenyl-2-picrylhydrazyl (DPPH), 2,2’-azino-bis (3-ethylbenzothiazoline-6-sulfonic acid (ABTS)), reducing power (cupric ion reducing activity (CUPRAC), ferric reducing antioxidant power (FRAP)), total antioxidant capacity (phosphomolybdenum (PHPD)) and metal chelating power using ferrous ions were conducted to evaluate antioxidant properties. Acetylcholinesterase (AChE), butyrylcholinesterase (BChE), α-amylase, α-glucosidase and tyrosinase were used to evaluate enzymatic inhibitory properties. 

Antioxidant abilities were evaluated as standard equivalents (trolox (TE, for ABTS, DPPH, FRAP, CUPRA and PHPD) and EDTA (EDTAE, for metal chelating)). Enzyme inhibitory assays results were expressed as IC_50_ values. Galantamine (GALAE, for cholinesterase), kojic acid (KAE, for tyrosinase), acarbose (ACAE, for amylase and glucosidase) were used as standard inhibitors in the enzyme assays. The detailed experimental procedures were given in the Supplementary Information as mentioned by our previous papers [38,39]. The detailed experimental procedures were given in the Supplementary File.

### 3.3. Profiling of Bioactive Metabolites Using Ultra Performance High-Pressure Liquid Chromatography (UHPLC) 

Dionex Ultimate 3000RS HPLC instrument was used to analyse the phytochemical composition of the extracts. Before RP-HPLC analysis, the extracts were filtered through 0.22 μm PTFE filter membrane (Labex Ltd., Hungary). The filtered samples were injected onto a Thermo Accucore C18 (100 mm × 2, i.d., 2.6 μm) column thermostated at 25 °C (±1 °C). The solvents used were water (A) and methanol (B). Both were acidified with 0.1% formic acid. The flow rate was maintained at 0.2 mL min^−1^. The elution gradient was isocratic 5% B (0–3 min), a linear gradient increasing from 5% B to 100% (3–43 min), 100% B (43–61 min), a linear gradient decreasing from 100% B to 5% (61–62 min) and 5% B (62–70 min). The column was coupled with a Thermo Q Exactive Orbitrap mass spectrometer (Thermo Scientific, USA) equipped with electrospray ionization source. Spectra were recorded in positive- and negative-ion mode, respectively, between *m*/*z* 100 and 1500.

The TraceFinder 3.1 (Thermo Scientific, USA) software was used for nontargeted screening. Most of the compounds were identified based on literature data and/or our previously published works. All cases, retention time, exact molecular mass, isotopic pattern, essential fragments with a given (5 ppm) mass tolerance were used for the identification of the compounds. Peaks that were detected in blank runs and those were also detected in the samples were rejected. Compounds which were confirmed by standards are marked in Table 2.

### 3.4. Statistical Analysis

All the data were given as mean ± SD and the statistical procedures were performed using R software v. 3.5.1. One-way ANOVA followed by Tukey’s multiple range was conducted to measure differences (*p* < 0.05) between the tested samples. Principal component analysis (PCA) and hierarchical cluster analysis (HCA) were performed to evaluate the differences of the tested seaweeds in terms of biological activities. 

## 4. Conclusions

The present study highlights for the first time the phytochemical profile, antioxidant capacities and enzyme inhibitory properties of five seaweeds. The seaweeds showed low to moderate antioxidant and enzymatic activities, with *A. anceps* showing the highest antioxidant properties, attributed to its high level of phenolics compounds. Hence, *A. anceps* could be considered as an effective source of natural antioxidants which deserves further consideration. This observation was further supported via multivariate analysis. *Halimeda* spp. was the least potent seaweed in terms of antioxidant and enzymatic properties but with potent anti-tyrosinase activity which needs further attention. The presence of loliolide compound in all of the five seaweeds warrants further investigations particularly in terms of cytotoxicity analysis and bioavailability. This study has established baseline data on these seaweeds which could be further explored for potential sustainable development of novel bioproducts.

## Figures and Tables

**Table 1:** Antioxidant properties of the tested seaweed extracts. *

Assays	*Halimeda* spp.	*Spyridia hypnoides*	*Valoniopsis pachynema*	*Gracilaria fergusonii*	*Amphiroa anceps*
DPPH (mg TE/g)	2.33 ± 0.04e	2.55 ± 0.05d	2.68 ± 0.03c	3.83 ± 0.09b	4.43 ± 0.07a
ABTS (mg TE/g)	5.36 ± 0.47e	8.91 ± 0.30d	11.27 ± 1.07c	13.26 ± 0.45b	18.56 ± 0.10a
CUPRAC (mg TE/g)	9.47 ± 0.17c	10.95 ± 0.20c	25.71 ± 0.73b	7.31 ± 0.07d	46.47 ± 1.60a
FRAP (mg TE/g)	4.91 ± 0.05d	6.27 ± 0.10c	9.03 ± 0.10b	5.24 ± 0.14d	13.99 ± 0.30a
PHPD (mmol TE/g)	0.21 ± 0.01c	0.37 ± 0.02b	0.30 ± 0.04bc	0.23 ± 0.01c	0.73 ± 0.06a
Chelating ability (mg EDTAE/g)	1.62 ± 0.15d	6.07 ± 0.56c	9.05 ± 0.76b	NA	16.99 ± 0.11a

* Values are expressed as mean ± S.D. EDTAE, EDTA equivalent; PHPD, phosphomolybdenum assay; NA, not active. Different letters indicate significant differences in the extracts (*p* < 0.05).

**Table 2:** Enzyme inhibitory effects (IC_50_ = mg/mL) of the tested seaweed extracts. *

Assays	AChE	BChE	Tyrosinase	Alpha-Amylase	Alpha-Glucosidase
*Halimeda* spp.	3.07 ± 0.10a	7.82 ± 0.67a	3.70 ± 0.06b	8.19 ± 0.23a	3.20 ± 0.31b
*Spyridia hypnoides*	3.18 ± 0.05a	7.96 ± 1.01a	3.73 ± 0.04b	7.31 ± 0.36b	4.11 ± 0.40a
*Valoniopsis pachynema*	3.25 ± 0.06a	8.75 ± 1.31a	3.68 ± 0.03b	7.02 ± 0.28b	2.57 ± 0.02c
*Gracilaria fergusonii*	3.27 ± 0.10a	7.43 ± 1.00a	3.70 ± 0.05b	8.27 ± 0.17a	4.90 ± 0.33a
*Amphiroa anceps*	3.90 ± 0.83a	6.68 ± 0.83a	4.49 ± 0.15a	5.34 ± 0.14c	5.64 ± 1.19a
Galantamine	0.003 ± 0.0001b	0.004 ± 0.0001b	NT	NT	NT
Kojic acid	NT	NT	0.09 ± 0.01c	NT	NT
Acarbose	NT	NT	NT	0.50 ± 0.01d	0.75 ± 0.02d

* Values are expressed as mean ± S.D. NT: not tested. Different letters indicate significant differences in the extracts (*p* < 0.05).

**Table 3:** Chemical composition of five seaweeds.

No.	Name	Formula	R*t*	[M + H]^+^	[M − H]^−^	Fragment 1	Fragment 2	Fragment 3	Fragment 4	Fragment 5
*Halimeda* spp.										
1	Acetylcholine	C_7_H_15_NO_2_	1.20	146.11810		87.0446	60.0816			
2	Unidentified hydroxycarboxylic acid	C_10_H_18_O_4_	13.50		201.11268	183.1017	57.1221	139.1116		
3	Hydroxyethylimino-phenylpropanol derivative	C_11_H_15_NO_2_	17.08	194.11810		176.1072	152.1071	134.0967	117.0702	91.0547
4	3-Methyladipic acid	C_7_H_12_O_4_	17.32		159.06574	141.0544	115.0750	97.0642		
5	Loliolide or isololiolide	C_11_H_16_O_3_	18.19	197.11777		179.1068	161.0962	135.1171	133.1014	107.0859
6	Loliolide or isololiolide	C_11_H_16_O_3_	19.47	197.11777		179.1069	161.0963	135.1170	133.1014	107.0860
7	Bromocarboxylic acid	C_7_H_5_BrO_3_	19.73		214.93438	170.9440	78.9173			
8	Caulerpin	C_24_H_18_N_2_O_4_	34.52	399.13449		385.1174	367.1080	340.1209	308.0944	280.0995
9	Unidentified alkaloid	C_24_H_18_N_2_O_4_	37.16	399.13449		363.1574				
*Spyridia hypnoides*										
1	Pantothenic acid	C_9_H_17_NO_5_	5.16	220.11850		202.1076	184.0971	174.1123	116.0346	90.0555
2	3-(4-Hydroxyphenyl) lactic acid	C_9_H_10_O_4_	8.93		181.05009	163.0392	135.0441	119.0491	72.9917	
3	Kynurenic acid isomer	C_10_H_7_NO_3_	14.34	190.05042		162.0550	144.0445	116.0498		
4	3-Phenyllactic acid	C_9_H_10_O_3_	16.84		165.05517	147.0438	119.0489	72.9915		
5	3-Methyladipic acid	C_7_H_12_O_4_	17.33		159.06574	141.0546	115.0750	97.0645		
6	Loliolide or isololiolide	C_11_H_16_O_3_	18.18	197.11777		179.1069	161.0962	135.1171	133.1014	107.0859
7	Riboflavin	C_17_H_20_N_4_O_6_	18.60	377.14611		359.1344	243.0879	200.0819	172.0869	99.0444
8	Loliolide or isololiolide	C_11_H_16_O_3_	19.46	197.11777		179.1069	161.0962	135.1171	133.1014	107.0860
9	N-(2-Phenylethyl) acetamide	C_10_H_13_NO	20.00	164.10754		122.0967	105.0703	90.9482	79.0548	
10	Lumichrome	C_12_H_10_N_4_O_2_	23.88	243.08821		216.0768	200.0825	198.0665	172.0871	
*Valoniopsis pachynema*										
1	Betaine	C_5_H_11_NO_2_	1.22	118.08681		59.0737	58.0659			
2	Ectoine	C_6_H_10_N_2_O_2_	1.22	143.08206		101.0715	97.0766	73.0768	68.0502	56.0502
3	Acetylcholine	C_7_H_15_NO_2_	1.23	146.11810		87.0446	60.0816			
4	Pantothenic acid	C_9_H_17_NO_5_	5.17	220.11850		202.1078	184.0972	174.1123	116.0347	90.0555
5	4-Hydroxybenzoic acid	C_7_H_6_O_3_	8.99		137.02387	93.0330	65.0382			
6	Kynurenic acid	C_10_H_7_NO_3_	13.02	190.05042		162.0551	144.0445	116.0495	89.0386	
7	Kynurenic acid isomer	C_10_H_7_NO_3_	14.33	190.05042		162.0549	144.0446	116.0496		
8	Methyladipic acid isomer	C_7_H_12_O_4_	15.04		159.06574	141.0544	115.0750	97.0644		
9	3-Phenyllactic acid	C_9_H_10_O_3_	16.82		165.05517	147.0440	119.0487	72.9914		
10	Loliolide or isololiolide	C_11_H_16_O_3_	18.19	197.11777		179.1070	161.0963	135.1171	133.1015	107.0860
11	Azelaamic acid (9-Amino-9-oxononanoic acid)	C_9_H_17_NO_3_	18.64		186.11302	125.0958	123.0803	97.0645		
12	Loliolide or isololiolide	C_11_H_16_O_3_	19.46	197.11777		179.1069	161.0962	135.1171	133.1015	107.0860
13	Chicoric acid (2,3-Di-*O*-caffeoyltartaric acid)	C_22_H_18_O_12_	19.53		473.07201	311.0414	293.0303	219.0298	179.0340	149.0080
14	N-(2-Phenylethyl) acetamide	C_10_H_13_NO	19.99	164.10754		122.0967	105.0704	90.9483	79.0549	
15	Hydroxycapric acid	C_10_H_20_O_3_	33.27		187.13342	141.1270	59.0123			
16	Caulerpin	C_24_H_18_N_2_O_4_	34.51	399.13449		385.1177	367.1078	340.1208	308.0943	280.0998
17	Hydroxyundecanoic acid isomer 1	C_11_H_22_O_3_	35.38		201.14907	59.0123				
18	Hydroxyundecanoic acid isomer 2	C_11_H_22_O_3_	35.87		201.14907	59.0123				
19	Hydroxydodecanoic acid	C_12_H_24_O_3_	38.04		215.16472	169.1581	59.0123			
*Gracilaria fergusonii*										
1	Gigartinine	C_7_H_15_N_5_O_3_	1.20	218.12532		133.0973	116.0709	115.0869	86.0354	70.0657
2	Phenethylamine	C_8_H_11_N	3.67	122.09698		105.0703	103.0548	79.0548		
3	Methyladipic acid isomer	C_7_H_12_O_4_	15.03		159.06574	141.0547	115.0750	97.0645		
4	3-Phenyllactic acid	C_9_H_10_O_3_	16.82		165.05517	147.0440	119.0488	72.9915		
5	Loliolide or isololiolide	C_11_H_16_O_3_	18.17	197.11777		179.1069	161.0961	135.1171	133.1014	107.0859
6	Loliolide or isololiolide	C_11_H_16_O_3_	19.46	197.11777		179.1069	161.0962	135.1170	133.1014	107.0860
7	Chicoric acid (2,3-Di-*O*-caffeoyltartaric acid)	C_22_H_18_O_12_	19.53		473.07201	311.0414	293.0303	219.0298	179.0340	149.0080
8	N-(2-Phenylethyl) acetamide	C_10_H_13_NO	19.98	164.10754		122.0967	105.0703	90.9482	79.0549	
9	Lumichrome	C_12_H_10_N_4_O_2_	23.88	243.08821		216.0772	200.0819	198.0671	172.0869	
10	Dihydrololiolide or dihydroisololiolide	C_11_H_18_O_3_	30.06	199.13342		181.1225	163.1118	153.1275	135.1170	107.0859
11	Hydroxydodecanoic acid	C_12_H_24_O_3_	38.04		215.1647	169.1595	59.0123			
*Amphiroa anceps*										
1	3-(4-Hydroxyphenyl) lactic acid	C_9_H_10_O_4_	9.01		181.05009	163.0386	135.0441	119.0487	72.9916	
2	Methyladipic acid isomer	C_7_H_12_O_4_	15.07		159.06574	141.0546	115.0750	97.0644		
3	N-Acetylisoleucine	C_8_H_15_NO_3_	15.92		172.09737	130.0860	128.1068			
4	N-Acetylleucine	C_8_H_15_NO_3_	16.75		172.09737	130.0860	128.1068			
5	3-Phenyllactic acid	C_9_H_10_O_3_	16.85		165.05517	147.0439	119.0489	72.9915		
6	Indoleacetic acid	C_10_H_9_NO_2_	17.26		174.05551	130.0649	128.0491			
7	3-Methyladipic acid	C_7_H_12_O_4_	17.36		159.06574	141.0547	115.0750	97.0644		
8	4-Coumaric acid	C_9_H_8_O_3_	17.72		163.03952	119.0488	93.0331			
9	Loliolide or isololiolide	C_11_H_16_O_3_	18.20	197.11777		179.1068	161.0961	135.1170	133.1015	107.0859
10	Riboflavin	C_17_H_20_N_4_O_6_	18.58	377.14611		359.1341	243.0876	200.0822	172.0866	99.0445
11	Indole carboxaldehyde	C_9_H_7_NO	18.91	146.06059		118.0654	117.0577	91.0547		
12	Loliolide or isololiolide	C_11_H_16_O_3_	19.47	197.11777		179.1068	161.0961	135.1170	133.1014	107.0859
13	Chicoric acid (2,3-Di-*O*-caffeoyltartaric acid)	C_22_H_18_O_12_	19.53		473.07201	311.0414	293.0303	219.0298	179.0340	149.0080
14	N-(2-Phenylethyl) acetamide	C_10_H_13_NO	20.00	164.10754		122.0967	105.0703	90.9482	79.0549	
15	Caffeoyl phenylethanoid glycoside isomer 1	C_29_H_36_O_15_	21.97		623.19760	161.0232	133.0280			
16	Caffeoyl phenylethanoid glycoside isomer 2	C_29_H_36_O_15_	23.22		623.19760	161.0232	133.0282			
17	Lumichrome	C_12_H_10_N_4_O_2_	23.85	243.08821		216.0768	200.0823	198.0660	172.0870	
18	Unidentified terpene 1	C_11_H_16_O_2_	26.59	181.12285		163.1119	145.1014	135.1171	121.1014	107.0859
19	Unidentified terpene 2	C_11_H_18_O_3_	30.06	199.13340		181.1224	163.1117	145.1013	135.1171	111.0443
20	Unidentified terpene 3	C_20_H_30_O_4_	32.47	335.22223		317.2114	299.2006	281.1903	273.1854	255.1740
21 ^1^	Eicosapentaenoic acid	C_20_H_30_O_2_	44.57		301.21676	257.2273	203.1801	135.1166		
22	Pheophytin A	C_55_H_74_N_4_O_5_	62.78	871.57375		593.2763	533.2549	460.2259		
23	Pheophytin A isomer	C_55_H_74_N_4_O_5_	64.99	871.57375		593.2764	533.2552	459.2172		

^1^ Confirmed by standard.

## Supplementary Materials

The following are available online at https://www.mdpi.com/1660-3397/18/4/198/s1, Figure S1: Extracted Ion Chromatogram (XIC) of compound 7 in *Halimeda* spp. at m/z 214.93438 (A) MS2 spectrum of compound 7 in *Halimeda* spp (B). Compound details available in Table 2. Figure S2: Extracted Ion Chromatogram (XIC) of compounds 10 and 12 in *Valoniopsis pachynema* at m/z 197.11777 (A). MS2 spectrum of compound 12 in *Valoniopsis pachynema* (B). Compounds details available in Table 2. Figure S3: Extracted Ion Chromatogram (XIC) of compounds 17 and 18 in *Valoniopsis pachynema* at m/z 201.14907 (A). MS2 spectrum of compound 18 in *Valoniopsis pachynema* (B). Compounds details available in Table 2. Figure S4: Extracted Ion Chromatogram (XIC) of compounds 20 in *Amphiroa anceps* at m/z 335.22223 (A). MS2 spectrum of compound 20 in *Amphiroa anceps* (B). Compounds details available in Table 2. The detailed experimental procedures for antioxidant and enzyme inhibitory bioassays were given in supplementary file.
