# Supplementary material for: In Vitro Enzyme Inhibitory Properties, Secondary Metabolite Profiles and Multivariate Analysis of Five Seaweeds
Source: Mar Drugs. 2020 Apr 8;18(4):198. doi: 10.3390/md18040198 (PMC7230894; doi:10.3390/md18040198)
Supplement: Supplementary file 1 [file marinedrugs-18-00198-s001.pdf]

In vitro enzyme inhibitory properties, secondary metabolites profiles and multivariate analysis of five seaweeds

Mohamad Fawzi Mahomoodally[1,2#], Nabeelah Bibi Sadeer[2#], Gokhan Zengin[3#], Zoltán Cziáky[4], József Jekő[4], Alina Diuzheva[5], Kouadio Ibrahime Sinan[3], Kishneth Palaniveloo[6], Doo Hwan Kim[7], Kannan RR Rengasamy[7#*]

[1]Institute of Research and Development, Duy Tan University, Da Nang 550000, Vietnam

[2]Department of Health Sciences; Faculty of Science, University of Mauritius, Réduit, Mauritius

[3]Department of Biology, Science Faculty, Selcuk University, Konya, Turkey

[4]Agricultural and Molecular Research and Service Institute, University of Nyíregyháza, Nyíregyháza, Hungary

[5]Department of Forest Protection and Entomology, Faculty of Forestry and Wood Sciences, Czech University of Life Sciences, Prague, Czech Republic

[6]Institute of Ocean and Earth Sciences, Institute for Advanced Studies Building, University of Malaya, Wilayah Persekutuan Kuala Lumpur 50603, Malaysia

[7]Department of Bioresources and Food Science, Konkuk University, Seoul 05029, South Korea

#These authors were contributed equally to this work

*Correspondence: Rengasamy@iceir.net; Tel.: +82-10-4297-2707 (KRRR)

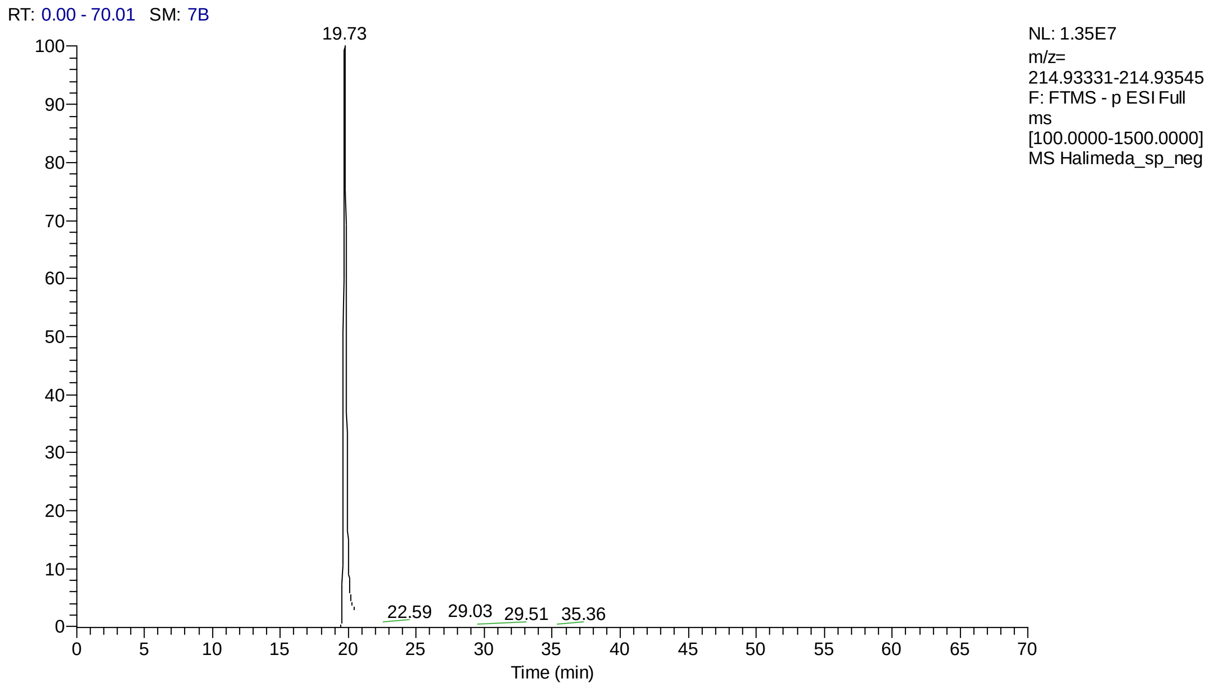

A

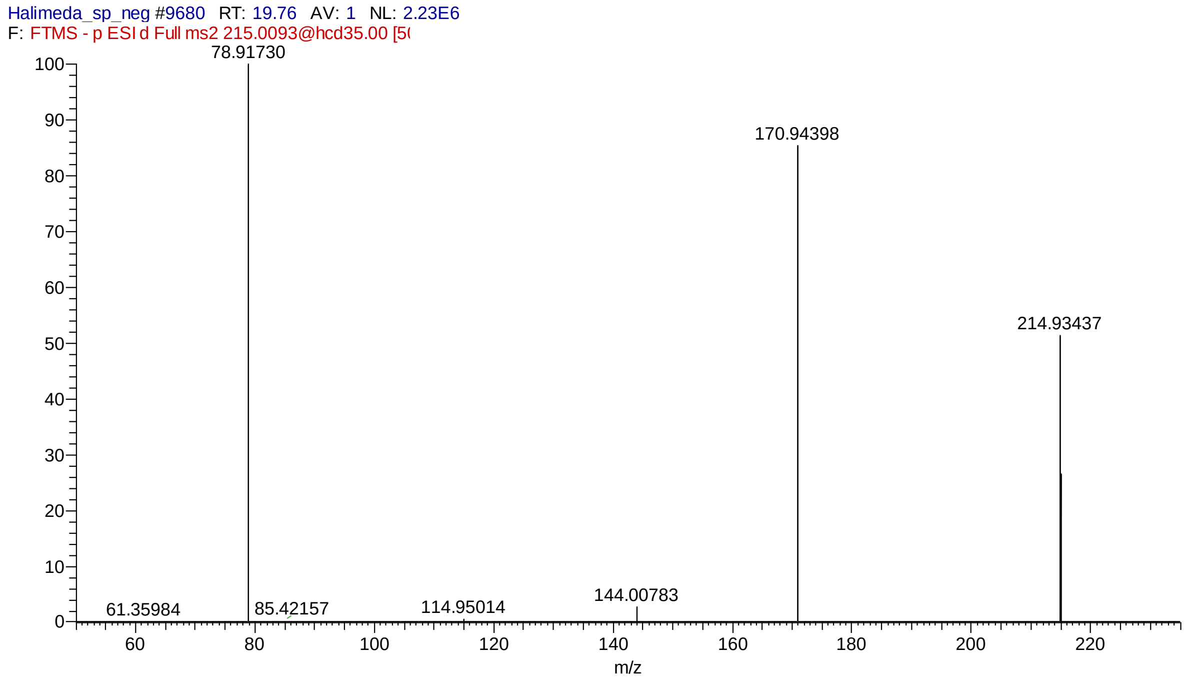

B

Figure S1. Extracted Ion Chromatogram (XIC) of compound 7 in Halimeda sp at *m/z* 214.93438 (A) MS2 spectrum of compound 7 in Halimeda sp (B). Compound details available in Table 2.

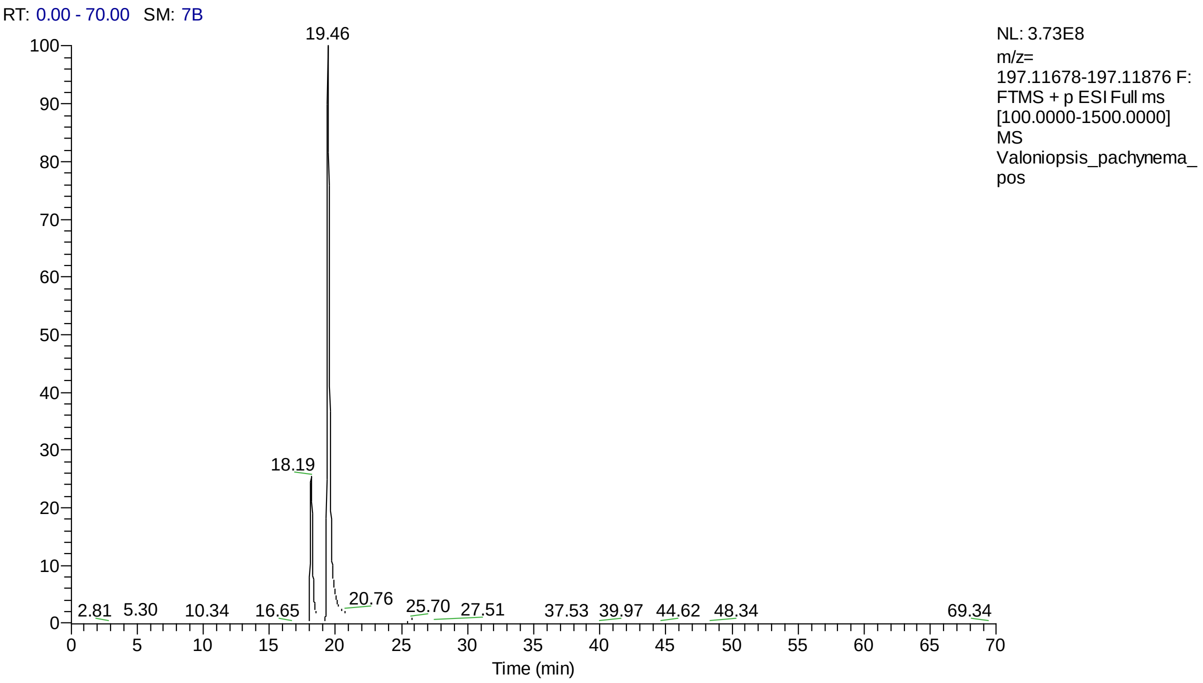

A

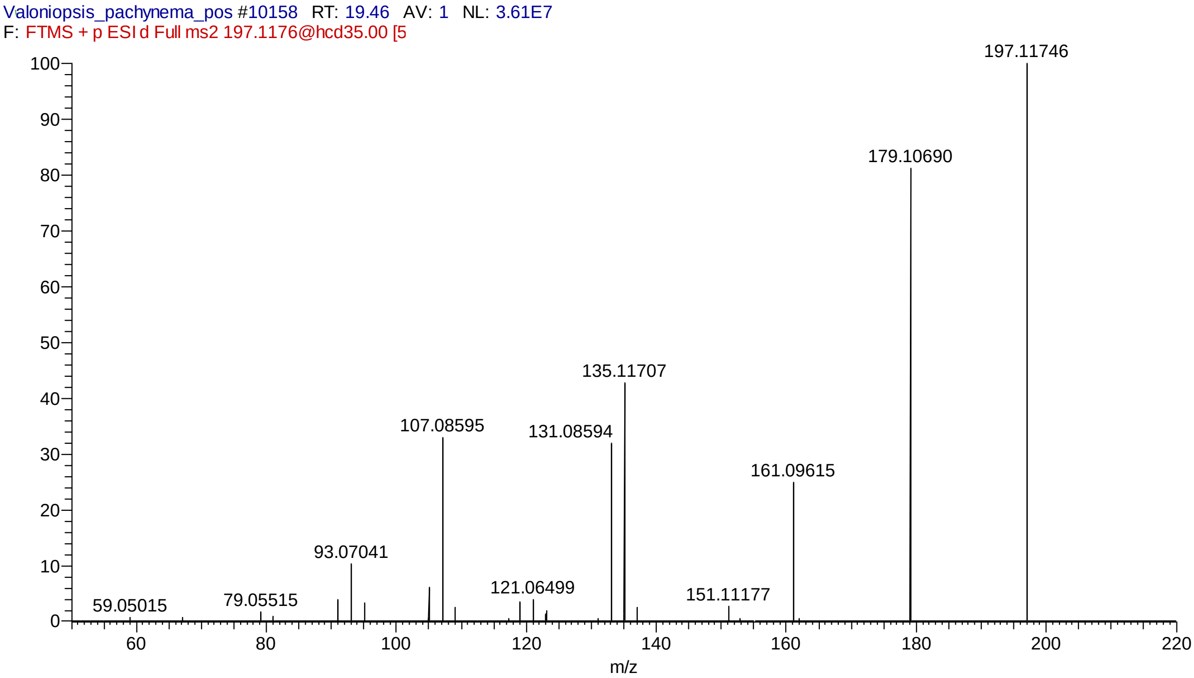

B

Figure S2. Extracted Ion Chromatogram (XIC) of compounds 10 and 12 in *Valoniopsis pachynema* at *m*/*z* 197.11777 (A). MS2 spectrum of compound 12 in *Valoniopsis pachynema* (B). Compounds details available in Table 2.

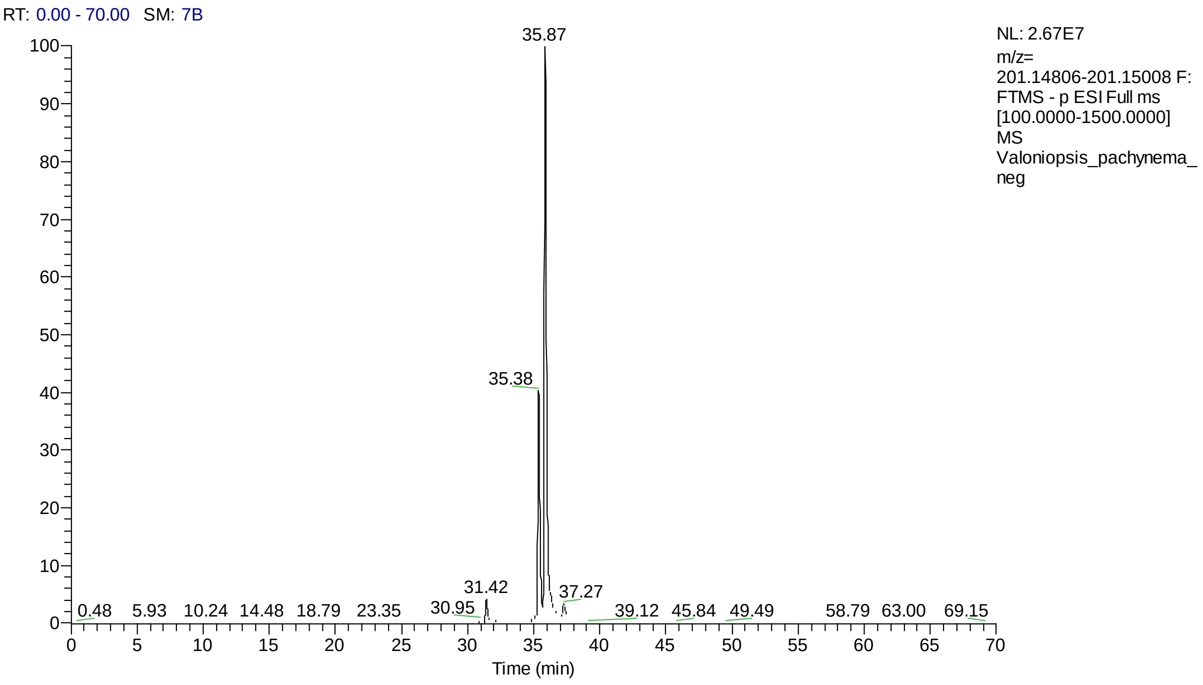

A

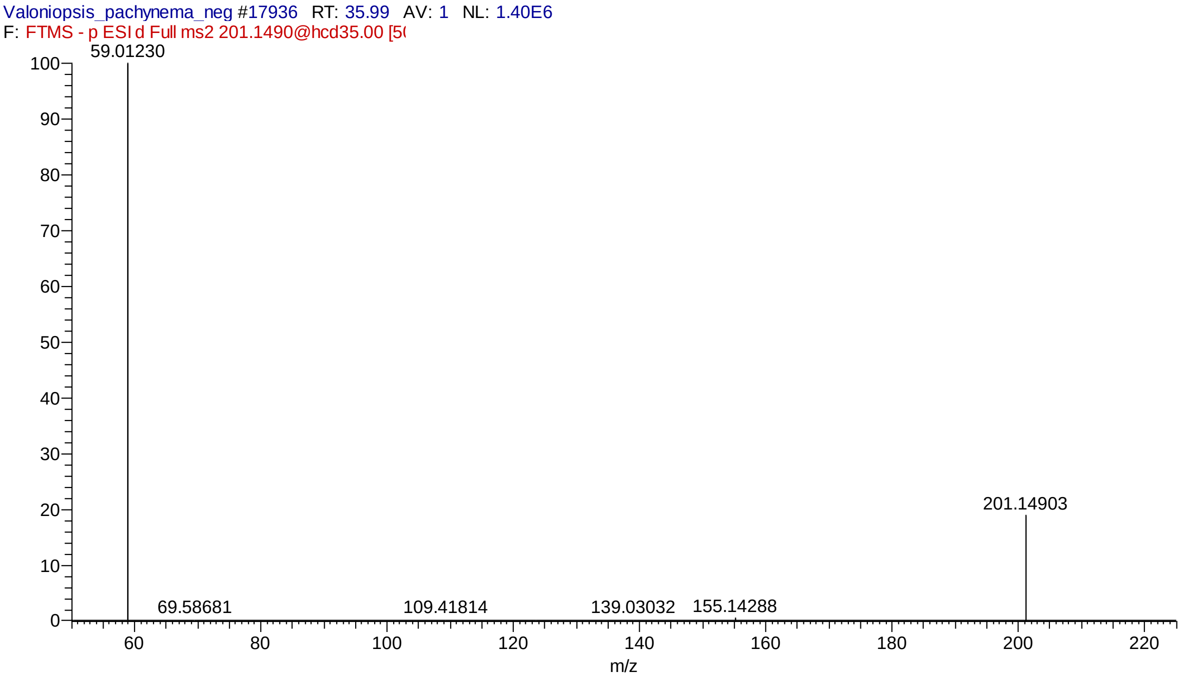

B

Figure S3. Extracted Ion Chromatogram (XIC) of compounds 17 and 18 in *Valoniopsis pachynema* at *m/z* 201.14907 (A). MS2 spectrum of compound 18 in *Valoniopsis pachynema* (B). Compounds details available in Table 2.

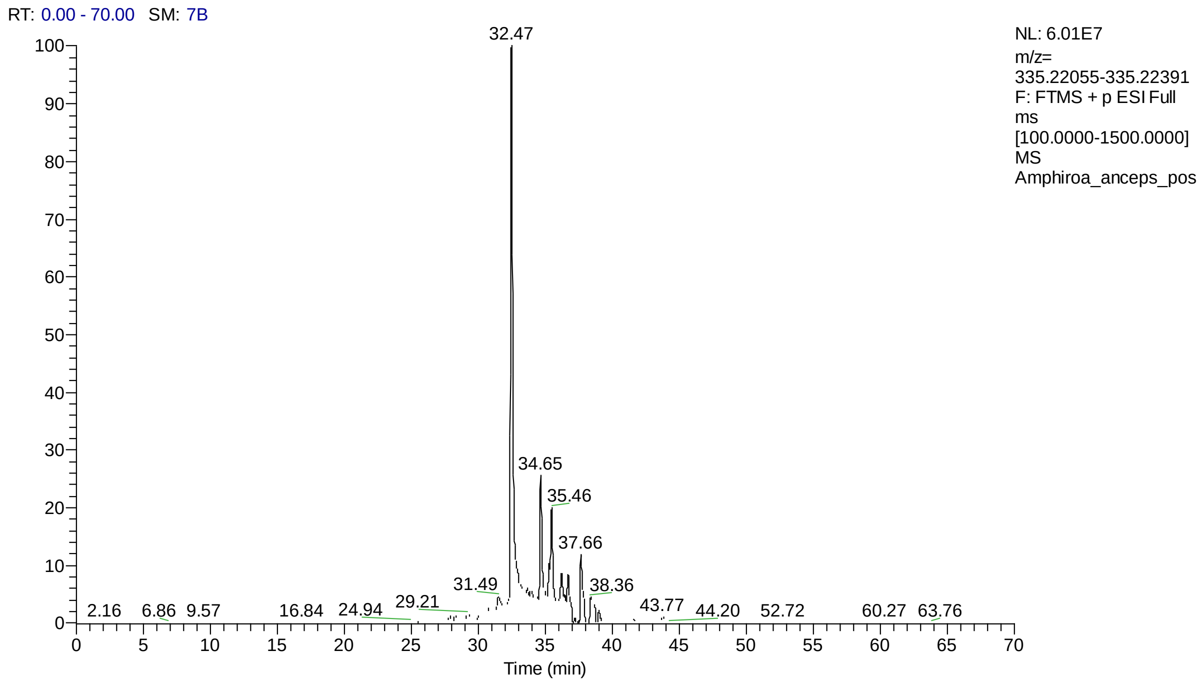

A

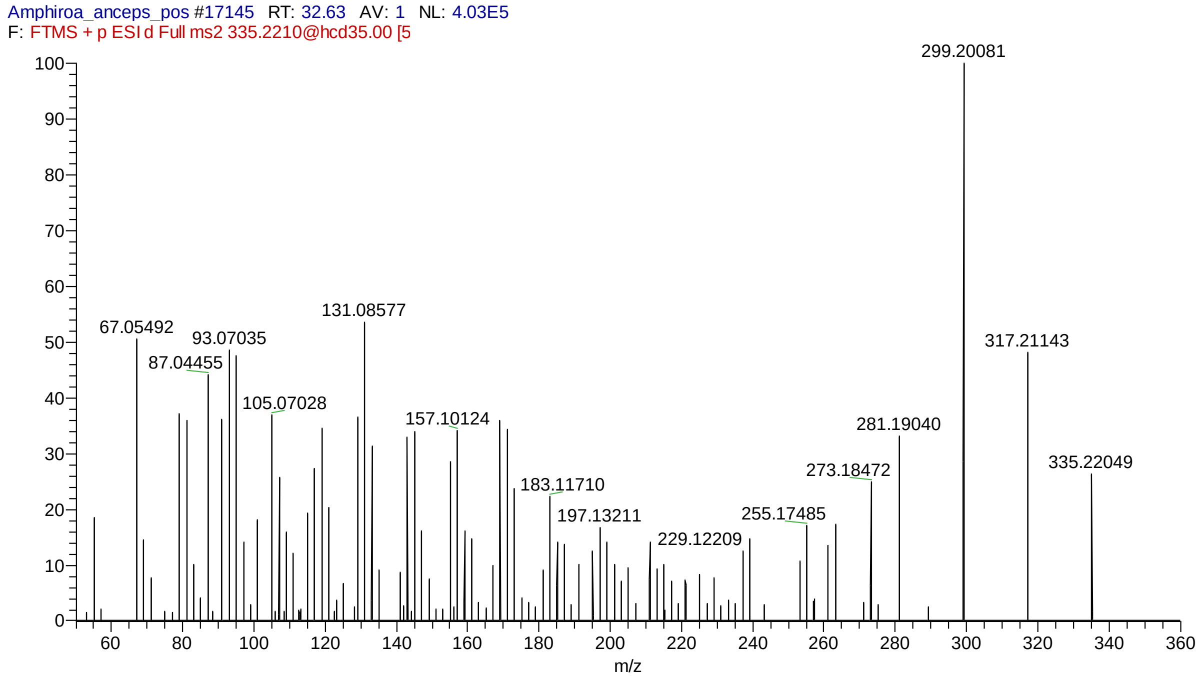

B

Figure S4. Extracted Ion Chromatogram (XIC) of compounds 20 in *Amphiroa anceps* at *m/z* 335.22223 (A). MS2 spectrum of compound 20 in *Amphiroa anceps* (B). Compounds details available in Table 2.

Materials and Methods

Determination of antioxidant and enzyme inhibitory effects

Free radical scavenging activity (DPPH)

The effect of the samples on 1,1-diphenyl-2-picrylhydrazyl (DPPH) radical was estimated according to Zengin and Aktumsek [38]. Sample solution (1 mL) was added to a 4 ml of a 0.004% methanol solution of DPPH. The sample absorbance was read at 517 nm after a 30 min incubation at room temperature in dark. The DPPH radical scavenging activity was expressed as equivalents of Trolox (mg TE/g).

ABTS (2,2 Azino-bis (3-ethylbenzothiazloine-6-sulfonic acid)) radical cation scavenging activity

The scavenging activity against ABTS cation radical was measured according to the method of Zengin and Aktumsek [38] with slight modification. Briefly, $ABTS^{\cdot+}$ radical cation was produced directly by reacting 7 mM ABTS solution with 2.45 mM potassium persulfate and allowing the mixture to stand for 12–16 in dark at the room temperature. Prior to beginning the assay, ABTS solution was diluted with methanol to an absorbance of 0.700±0.02 at 734 nm. Sample solution (1 mL) was added to ABTS solution (2 mL) and mixed. The sample absorbance was read at 734 nm after a 30 min incubation at room temperature. The ABTS radical cation scavenging activity was expressed as equivalents of Trolox (mg TE/g).

Cupric ion reducing (CUPRAC) method

The cupric ion reducing activity (CUPRAC) was determined according to the method of Zengin and Aktumsek [38]. Sample solution (0.5 mL) was added to premixed reaction mixture containing $CuCl_2$ (1 mL, 10 mM), neocuproine (1 mL, 7.5 mM in ethanol) and NH_4Ac buffer (1 mL, 1 M, pH 7.0). Similarly, a blank was prepared by adding sample solution (0.5 mL) to premixed reaction mixture (3 mL) without $CuCl_2$. Then, the sample and blank absorbances were read at 450 nm after a 30 min incubation at room temperature. The absorbance of the blank was subtracted from that of the sample. CUPRAC activity was expressed as equivalents of Trolox (mg TE/g).

Ferric reducing antioxidant power (FRAP) method

The FRAP assay was carried out as described by Zengin and Aktumsek [38] with slight modification. Sample solution (0.1 mL) was added to premixed FRAP reagent (2 mL) containing acetate buffer (0.3 M, pH 3.6), 2,4,6-tris(2-pyridyl)-s-triazine (TPTZ) (10 mM) in 40 mM HCl and ferric chloride (20 mM) in a ratio of 10:1:1 (v/v/v). The sample absorbance was read at 593 nm after a 30 min incubation at room temperature. FRAP activity was expressed as equivalents of Trolox (mg TE/g)

Phosphomolybdenum method

The total antioxidant activity of the samples was evaluated by phosphomolybdenum method according to Zengin and Aktumsek [38] with slight modification. Sample solution (0.3 mL) was combined with 3 mL of reagent solution (0.6 M sulfuric acid, 28 mM sodium phosphate and 4 mM ammonium molybdate). The sample absorbance was read at 695 nm after a 90 min incubation at 95 °C. The total antioxidant capacity was expressed as equivalents of Trolox (mg TE/g).

Metal chelating activity on ferrous ions

The metal chelating activity on ferrous ions was determined by the method described by Zengin and Aktumsek [38]. Briefly, sample solution (2 mL) was added to $FeCl_2$ solution (0.05 mL, 2 mM). The reaction was initiated by the addition of 5 mM ferrozine (0.2 mL). Similarly, a blank was prepared by adding sample solution (2 mL) to $FeCl_2$ solution (0.05 mL, 2 mM) and water (0.2 mL) without ferrozine. The sample and blank absorbances were read at 562 nm after 10 min incubation at room temperature.

The absorbance of the blank was subtracted from that of the sample. The metal chelating activity was expressed as equivalents of EDTA (mg EDTA/g).

Cholinesterase inhibitory activity

Cholinesterase (ChE) inhibitory activity was measured using Ellman's method, as previously reported by Zengin and Aktumsek [38]. To 50 μL sample solution, 125 μL Ellman reagent (DTNB) and 25 μL AChE (or BChE) solution prepared in Tris-HCl buffer (pH 8.0) were added in a 96-well microplate and allowed to incubate for 15 min at room temperature. The reaction was started with the addition of 25 μL acetylthiocholine iodide (ATCI) or butyrylthiocholine chloride (BTCl). In the same way, a blank was prepared by adding sample solution to all reaction reagents except the enzyme solution (AChE or BChE). The sample and blank absorbance were read at 405 nm after 10 min incubation at room temperature. To obtain real absorbance, the blank absorbance was subtracted from sample absorbance. The cholinesterase inhibitory activity was expressed Enzyme inhibitory assays results were expressed as IC50 value and galantamine was used as standard inhibitor.

Tyrosinase inhibitory activity

Tyrosinase inhibitory activity was measured using the modified dopachrome method using L-DOPA as substrate according to Zengin and Aktumsek [38]. To 25 μL sample solution, 40 μL tyrosinase solution was mixed followed by the addition of 100 μL phosphate buffer (pH 6.8) in a 96-well microplate allowed to incubate for 15 min at room temperature. The reaction was initiated by adding 40 μL of L-DOPA. Similarly, a blank was prepared by adding sample solution to all reaction reagents except the enzyme solution (tyrosinase). The sample and blank absorbances were read at 492 nm after a 10 min incubation at room temperature. To obtain real absorbance, the blank absorbance was subtracted from sample absorbance. The tyrosinase inhibitory activity was expressed Enzyme inhibitory assays results were expressed as IC50 value and kojic acid was used as standard inhibitor.

α-Amylase inhibitory activity

α-Amylase inhibitory activity was performed using Caraway-Somogyi iodine/potassium iodide (IKI) method Zengin and Aktumsek [38]. Briefly, 25 μL sample solution was mixed with 50 μL α-amylase solution in phosphate buffer (pH 6.9 with 6 mM sodium chloride) in a 96-well microplate and allowed to incubate for 10 min at 37 °C. After pre-incubation, initiation of the reaction was done by adding 50 μL starch solution (0.05%). Likewise, a blank was prepared by adding sample solution to all reaction reagents without enzyme solution (α-amylase). The reaction was then stopped by adding 25 μL HCl (1 M) followed by the addition of 100 μL iodine-potassium iodide solution. The sample and blank absorbances were read at 630 nm. To obtain real absorbance, the blank absorbance was subtracted from sample absorbance. The α-amylase inhibitory activity was expressed Enzyme inhibitory assays results were expressed as IC50 value and acarbose was used as standard inhibitor.

α-Glucosidase inhibitory activity

α-Glucosidase inhibitory activity was performed using the method as described by Zengin and Aktumsek [38]. Briefly, 50 μL sample solution was mixed with 50 μL glutathione, 50 μL α-glucosidase solution in phosphate buffer (pH 6.8) and 50 μL of 4-Nitrophenyl-β-D- glucopyranoside (PNPG) in a 96-well microplate and incubated for 15 min at 37 °C. Similarly, a blank was prepared by adding sample solution to all reaction reagents without enzyme solution (α-glucosidase). The reaction was stopped by adding 50 μL sodium carbonate (0.2 M). The sample and blank absorbances were read at 400 nm. To obtain real absorbance, the blank absorbance was subtracted from sample absorbance. Enzyme inhibitory assays results were expressed as IC50 value and acarbose was used as standard inhibitor.
